# The genetics of phenotypic plasticity. XII. Temporal and spatial heterogeneity

**DOI:** 10.1002/ece3.792

**Published:** 2013-10-22

**Authors:** Samuel M Scheiner

**Affiliations:** Division of Environmental Biology, National Science Foundation4201 Wilson Blvd., Arlington, VA 22230

**Keywords:** Developmental constraint, island migration, model, stepping-stone migration, theory

## Abstract

To understand empirical patterns of phenotypic plasticity, we need to explore the complexities of environmental heterogeneity and how it interacts with cue reliability. I consider both temporal and spatial variation separately and in combination, the timing of temporal variation relative to development, the timing of movement relative to selection, and two different patterns of movement: stepping-stone and island. Among-generation temporal heterogeneity favors plasticity, while within-generation heterogeneity can result in cue unreliability. In general, spatial variation more strongly favors plasticity than temporal variation, and island migration more strongly favors plasticity than stepping-stone migration. Negative correlations among environments between the time of development and selection can result in seemingly maladaptive reaction norms. The effects of higher dispersal rates depend on the life history stage when dispersal occurs and the pattern of environmental heterogeneity. Thus, patterns of environmental heterogeneity can be complex and can interact in unforeseen ways to affect cue reliability. Proper interpretation of patterns of trait plasticity requires consideration of the ecology and biology of the organism. More information on actual cue reliability and the ecological and developmental context of trait plasticity is needed.

## Introduction

We are faced with a paradox. Simple logic tells us that if organisms could always express the phenotype that corresponded to maximum fitness, such individuals should be favored by natural selection. Yet in most instances, instead of such ubiquitous phenotypic plasticity, most species differentiate into individuals with a range of fixed phenotypes (Hereford 2009). This paper is the latest in a recent series (Scheiner and Holt 2012; Scheiner et al. 2012) aimed at resolving that paradox by systematically exploring the propositions of the theory of the evolution of phenotypic plasticity (Appendix). [I urge reading those papers prior to this current missive as the results presented here build on those.] One way to categorize the factors that limit optimal plasticity are those that are external to the organism – patterns of environmental variability perceived by an organism due either to changes in its environment or to its movement – and those that are internal to an organism – costs of plasticity and developmental limitations. This paper addresses external factors. The purpose of this paper is not to provide definitive answers about when plasticity is and is not favored by selection. As you will see, the answer is complex. Rather, the purpose is to explore those complexities and make obvious the range of information that is necessary if empirical patterns are to be properly understood. The model explored here is meant to be general; as such it probes the boundaries of parameter space rather than attempting to provide precise predictions of a specific system.

Selection for phenotypic plasticity requires environmental heterogeneity (theory proposition 1). However, that simple statement belies much complexity, especially with regard to how different patterns of environmental heterogeneity affect cue reliability (proposition 4). The goal of this paper is to unpack that first proposition and examine many different ways that the environment can vary in space and time, to explore how that variability interacts with cue reliability, and how those different patterns of variation and interaction can favor or disfavor adaptation by phenotypic plasticity.

This exploration of the effect of environmental heterogeneity on the evolution of plasticity will sharpen our tests of the theory of the evolution of plasticity. Unfortunately, because of logistical difficulties and complexities, no strong tests of this theory have been performed, by which I mean precise quantitative predictions derived from appropriately designed models. At best, we have comparisons of empirical results with general, qualitative predictions (DeWitt and Scheiner 2004). By better defining types of environmental heterogeneity and exploring the resulting evolutionary outcomes, we can build more precise models and better link theory and data.

### Patterns of environmental heterogeneity

The environment can vary in time and/or space. That statement is both simple and obvious, yet hides much complexity. With respect to the evolution of plasticity, all variation must be considered relative to when the phenotype is determined, movement occurs, and selection happens. The complexities considered in this paper go well beyond previous models. Adding such complexities is more than an intellectual exercise as I show how these factors can interact in unexpected ways. Empirical studies very frequently fail to adequately describe their environmental context. Thus, an important task of this paper is to provide an overall framework for describing environmental heterogeneity and then to explore a substantial portion of that parameter space. Any given study will occupy a small portion of that space. However, with my framework and model outcomes, that study can be compared with others, and differences and similarities in patterns of plasticity can be understood.

In the model explored here, phenotypic determination is treated as a developmental stage such that the phenotype is fixed at some point in the life history. For some versions of my model, selection occurs immediately after development. Such a scenario can be conceived as selection on a labile trait in which selection occurs once during the organism's life. My model does not examine selection on continuously labile traits in which fitness is summed over multiple trait values. For such traits, as the rate of phenotypic change approaches instantaneous, the constraints on trait evolution become confined to factors that are internal to the organism (e.g., physiological system limitations). Consideration of such factors is outside the scope of this paper.

Assuming that an organism's phenotype is fixed following development, we can define two scales of temporal variation: among-generation and within-generation with change in the environment happening before or after development. Similarly, if an organism moves once during its life and selection occurs at a single instance, we can define two life history patterns: selection then movement and movement then selection. These two patterns, along with the presence or absence of spatial heterogeneity, define the types of environmental heterogeneity that are relevant to the evolution of phenotypic plasticity.

Temporal heterogeneity only, by definition, means that the population exists in a single, uniform deme with no movement among demes. For this scenario, there are two possible patterns of variation: change once per generation before development and change both before and after development but before selection. Those changes may be independent or be correlated. An example of such a pattern of change is the timing of snow melt in the spring determining the environment prior to development and late-summer rainfall determining the environment of selection. Change occurring after development only is not relevant because there must be environmental heterogeneity that affects phenotypic determination (theory proposition 1).

Expanding our consideration from a single deme to multiple demes now allows for the addition of spatial heterogeneity. My model has a fixed, underlying spatial pattern of heterogeneity. That heterogeneity is overlaid by temporal variation within demes such that the spatial pattern is a central tendency, that is, present but varying in magnitude and pattern.

We can define two patterns of spatial variation that represent the ends of a spectrum: gradient and mosaic. In the simplest case, a gradient is a one-dimensional, monotonic change such that distance in space is equivalent to difference in the environment. We can conceive of more complex patterns, such as two-dimensional gradients – or even a three-dimensional gradient in an aquatic environment – and nonmonotonic patterns of change. Or there may be some degree of spatial autocorrelation among demes resulting in an irregular pattern of similarity with distance. At the other extreme, a mosaic pattern of heterogeneity assumes a spatial autocorrelation of zero. My model examines the simplest case of a one-dimensional, linear gradient. In the real world, there is likely to be some amount of spatial autocorrelation in the environment (Urban 2011) with a linear gradient representing the central tendency of that correlation.

These patterns of spatial variation are mirrored by patterns of movement. Evolutionary models typically consider two patterns: stepping-stone migration and island migration. For stepping-stone migration, movement occurs between adjacent demes only or perhaps more distant demes with the probability of movement decreasing with distance. In contrast, for island migration, movement occurs among all demes with equal probability, although more complex movement rules are possible. Thus, from the perspective of an organism, an island migration pattern is equivalent to a mosaic spatial pattern. My model primarily explores the effects of a stepping-stone migration pattern, with some consideration of the island migration pattern.

Discussions of the effects of environmental heterogeneity on the evolution of plasticity often assume an equivalence between temporal variation after development and movement after development. As will be seen in this paper, variation in time does not lead to the same outcomes as variation in space, and their interaction can lead to complex patterns of evolutionary response.

The types and causes of environmental heterogeneity just described can be combined in a variety of ways. In this paper, I ignore subtleties and complexities of patterns of heterogeneity such that the various combinations represent the boundaries of possibilities. I consider three broad patterns that combine spatial and temporal variation (Table 1). In all cases, the environment varies in space along a gradient.

**Table 1:** Temporal patterns of environmental variation used in various simulations, including the life history stage at which that variation occurred and whether that variation was correlated within a single generation. Across space, the temporal variation could occur independently in each deme, or could be synchronized across all demes

	Life history stage		
		
Pattern	Before development	Before selection	Correlation
1	Fixed	Variable	0
2	Variable	Fixed	0
3	Variable	Variable	0–1

In the first pattern, the environment of development is fixed among generations, while the environment of selection varies among generations. An example of such a pattern would be plasticity in leaf traits where soil nutrient content determines leaf thickness, and fitness is later determined by temperature and precipitation.

In the second pattern, the environment varies among generations prior to development, while the environment of selection is fixed. An example would be plasticity in the adult size of dragonflies. The larval pond environment determines size at metamorphosis, which might vary from year to year in food availability; size then determines adult survival.

In the third pattern, the environment changes both prior to development and prior to selection. Those changes may or may not be correlated. For example, adult survival of dragonflies might depend on the interaction of size and temperature, with that temperature variation being independent of the conditions that determine larval food availability. In a contrasting example, leaf thickness might be determined by early-summer temperature and precipitation, and fitness determined by late-summer temperature and precipitation, with those climatic variables correlated with each other.

In the most extreme case, environmental change prior to development would carry over to the environment at selection, a correlation of one. For example, in the water flea, *Daphnia*, in the presence of predators, some species develop extended morphologies – head and tail elongations – that decrease predation (e.g., Krueger and Dodson 1981). *Daphnia* have generation times on the order of weeks, so that predator densities may vary among generations but be relatively constant within a generation.

Temporal variation might be synchronized in space. For example, mean temperature decreases with increasing elevation. In a warmer than average summer, an entire mountainside is likely to experience higher temperatures while maintaining that elevational gradient.

### Previous models of plasticity evolution

The comprehensive exploration of environmental heterogeneity presented in this paper has not been carried out by previous models (Berrigan and Scheiner 2004). All previous models examined temporal and spatial variation separately and as I will show, combining temporal and spatial variation leads to outcomes that are not predictable by either alone. Except for the recent papers in this series (Scheiner and Holt 2012; Scheiner et al. 2012), no model examined the interaction of spatial and temporal variation. Regarding spatial variation, nearly all models considered either only two demes or a spatial mosaic. In models that considered temporal variation, nearly all models had change occurring just once a generation. Most models assumed no change in the environment between when the phenotype is determined and when selection occurs, if they even made explicit development as a separate life history stage. With regard to patterns of movement, nearly all models assumed one of two patterns: a propagule pool that dispersed equally among all demes, or an island migration pattern with dispersal rates less than 100%. Often these movement patterns were only implied.

There are a few notable exceptions to those generalities. The models of de Jong and collaborators (De Jong 1999; Sasaki and De Jong 1999; De Jong and Behera 2002) and Gomulkeiwicz and Kirkpatrick (Gomulkiewicz and Kirkpatrick 1992) included environmental change after development. The models of Chevin and Lande (2011) and Scheiner (1998) assumed a gradient with stepping-stone migration. However, each of those models examined just a single aspect of environmental heterogeneity. As will be seen in this paper, the typical assumptions in models of plasticity evolution – among-generation temporal variation, few demes, an island migration pattern – are those that maximize selection for plasticity. More complex models are needed if we are to understand why adaptive plasticity is less common than we would expect.

## Model Structure

The model was an individual-based simulation (summary of parameters in Table 2) using a gene-based model of adaptation to an environmental gradient. It assumed that in the absence of temporal variation, the optimal phenotype changes in a linear fashion along that gradient, and the phenotypes of individuals can be expressed by a linear reaction norm. Gene expression is either responsive to the environment (plastic loci) or not (nonplastic loci). Adaptation can occur by two routes: genetic differentiation in which the allelic values of the plastic loci go to zero (i.e., are not expressed) or phenotypic plasticity in which the allelic values of the nonplastic loci go to zero. Because the optimal phenotype changes in a linear fashion along the gradient, and the environmental responsiveness of the plastic loci is linear, the plasticity optimum (where the realized trait value in each habitat is at the local optimum) is a possible outcome. Although presented as a dichotomy, intermediate outcomes are possible in which individuals express the optimal phenotype in a particular environment through nonzero values of both the plastic and nonplastic loci.

**Table 2:** Summary of the model parameters

Fixed parameters
Number of nonplastic and plastic loci = 5 each
Length of the environmental gradient = 50 demes
Steepness of the gradient (change in optimum in adjacent demes) = 0.4 units
Strength of selection within demes (σ) = 2 units
Population size = 100 individuals/deme
Number of generations = 10,000
Parameters explored
Life history pattern: selection before dispersal versus dispersal before selection
Timing of environmental change: before versus after development
Magnitude of environmental change
Correlation of environmental change within and among generations
Migration pattern: stepping-stone versus island
Dispersal rate

The model was implemented in Fortran 77 (the computer code is available from Dryad). The metapopulation consisted of a linear array of 50 demes. An environmental gradient was created by varying the optimal value of a single trait (phenotype) in a linear fashion along the array from −9.8 to +9.8 arbitrary units at the ends of the gradient, that is, the optimal phenotype in adjacent demes differed by 0.4 units. An individual's phenotype (trait value) was determined by 10 diploid loci: five plastic loci and five nonplastic loci. The loci contributed additively to the trait. Allelic values at the plastic loci were multiplied by an environment-dependent quantity before summing all allelic values. The effect of the environment in a particular deme (*E*_*i*_ for deme *i*) on the phenotypic contribution of each unit plastic allelic value varied in a linear fashion, with a slope of 0.04 units [*E*_*i*_ = 0.04(*i*−25.5)]. The phenotype of each individual was determined at the time of development as:


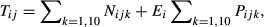
(1)

where *T*_*ij*_ is the phenotype of the *j*th individual that develops in the *i*th environment (deme), *N*_*ijk*_ is the allelic value of the *k*th nonplastic allele of that individual, and *P*_*ijk*_ is the allelic value of the *k*th plastic allele. There was no random component of phenotypic variation. For a given genotype, Σ*N*_*ijk*_ can also be thought of as the intercept of its reaction norm at the midpoint of the gradient, or the phenotype of the individual in the absence of plasticity, and [slope(*E*_*i*_)Σ*P*_*ijk*_] can be thought of as the slope of its reaction norm.

Life history events occurred in one of two sequences: (1) birth, followed by development (i.e., the phase in the life cycle when the phenotype is determined), then dispersal, selection, and reproduction (denoted as “move first”); or alternatively, (2) birth, development, selection, dispersal, and then reproduction (denote as “select first”). Selection was based on survival with the probability of surviving being a Gaussian function of the difference between an individual's phenotype and the locally optimal phenotype. Fitness (the probability of surviving) was determined as:


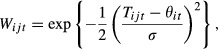
(2)

where *W*_*ij*_ is the fitness of the *j*th individual undergoing selection in the *i*th environment, *T*_*ij*_ is the phenotype of that individual, *θ*_*i*_ is the optimal phenotype in that environment, σ is the strength of selection (selection weakens as σ increases). Survival was determined by choosing a random number from a uniform distribution [0,1], and the individual died if its fitness was less than that value.

Temporal variation occurred at one or both of two life history stages: after reproduction but before development or after development before selection (Table 1). Depending on the simulation, this variation occurred once per generation at one of the two stages with the environment remaining fixed at the other stage, or occurred at both stages. If the variation occurred at both stages, those changes could be independent or they could be correlated, including a correlation of 100% (i.e., a single change that carried through the entire life cycle). Finally, the changes could be independent among demes (most simulations), or be synchronized among demes. Previous papers (Scheiner and Holt 2012; Scheiner et al. 2012) explored pattern 1 only.

Temporal autocorrelation was simulated using the recursion:


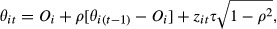
(3)

where *θ*_*it*_ is the environment at either development or selection in the *i*th deme in generation *t*, *O*_*i*_ is the mean or fixed environment in the *i*th deme (a linear function of *i*), τ is the standard deviation of environmental variation, ρ is the temporal autocorrelation coefficient, and *z*_*it*_ is a sequence of independent zero-mean, unit-variance Gaussian random deviates. For simulations without temporal variation, *τ* = 0, and for uncorrelated temporal variation, *ρ* = 0. The standard deviation of environmental noise (*τ*) is shown as a percentage of the difference in the optima at the two ends of the gradient. The autocorrelation (*ρ*) varied from −75 to 75%.

Dispersal occurred in one of two patterns: stepping-stone or island. For the stepping-stone migration pattern, the dispersal probability and the distance moved were determined using a zero-mean Gaussian random number, so that the probability of moving and the average distance moved were correlated (Scheiner and Holt 2012). Increasing the dispersal probability was carried out by increasing the variance of the Gaussian so that both more individuals were likely to move, and they were likely to move farther. Individuals that would otherwise migrate beyond the end of the gradient migrated to the terminal demes. For the island migration pattern, each individual had a fixed probability of moving. If it moved, it had an equal probability of moving to any of the other demes. For both patterns, dispersal per se had no cost; survival during dispersal was 100%.

Reproduction occurred following viability selection and was accomplished by assembling pairs of individuals within a deme at random with replacement, with each pair producing 1 offspring, then repeating until the carrying capacity of that deme was reached (100 individuals per deme). This procedure assumes soft selection in that local population size was determined independently of the outcome of selection. It also assumes that the spatial scale of reproduction and mating matches that of density dependence and the grain of the selective environment.

Each simulation was initialized with 100 individuals being born in each deme, or 1000 individuals for simulations with just temporal variation in a single deme. For each individual in the initial generation, allelic values (for both plastic and nonplastic loci) were chosen independently from the values −2, −1, 0, 1, and 2, with each value being equally likely. Even though initial values are discrete, due to mutation allelic values are continuous variables after the initial generation. When new offspring were generated, each allele mutated with a probability of 10%. [Lower mutation rates mainly changed the time-scale over which evolution occurs, rather than the eventual outcome (Scheiner and Holt 2012)]. When a mutation occurred, the allelic value was changed by adding a Gaussian deviate (mean of zero and a standard deviation of 0.1 units) to the previous allelic value (i.e., this is an infinite-alleles model).

All simulations were run for 10,000 generations to ensure that the equilibrium point (the point after which all calculated quantities showed no further directional trend) was reached (Scheiner and Holt 2012). Each parameter combination was replicated 20 times and the results shown are the means of those replicates. Coefficients of variation of reported parameters were generally low (5–20%). If the metapopulation went extinct, additional realizations were run until 20 successful replications were achieved; for some parameter combinations (see results), the extinction probability was 100% (i.e., no successful replications in 60 runs). Reported outcomes were averaged over successful replications only.

The reaction norm is a mathematical function describing how the phenotypic expression of a given genotype varies among environments. The plasticity of a linear reaction norm is best described by the slope of the function. In this model, the slope of the reaction norm is the product of the slope of *E*_*i*_ and the sum of the values of the plasticity alleles (i.e., the right-hand sum in eq. 1). For these simulations, as the slope of *E*_*i*_ was identical, the final outcome was measured as the average across all demes of the sum of the values of the plasticity alleles for each individual. That is, 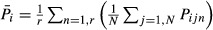, where 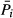 is the mean plasticity of the *i*th deme over all *r* runs, *N* = 100 is the number of individuals per deme, and *P*_*ijn*_ is the sum of the values of the plasticity alleles of the *j*th individual developing in the *i*th deme in the *n*th run. The overall mean plasticity 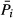 is the average of 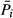 across demes and is given by 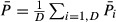, where *D* is the number of demes. [The order of averaging, over runs within demes first or over demes within runs first, does not affect the final average, because the number of demes is the same for all runs. Mean plasticity was calculated at each generation.] The average plasticity was standardized to the optimal reaction norm (i.e., relative plasticity) so that a pure plasticity outcome would have a value of 1 and a pure differentiation outcome would have a value of 0 (flat reaction norms). Intermediate values indicate that the average reaction norm had a slope intermediate between the two pure outcomes. Values outside this range were possible; that is, it was possible to achieve a reaction norm with a slope steeper than the optimal value (>1) or in a direction opposite from the optimal value (<0).

## Results

### Temporal variation only

**Figure 1:**
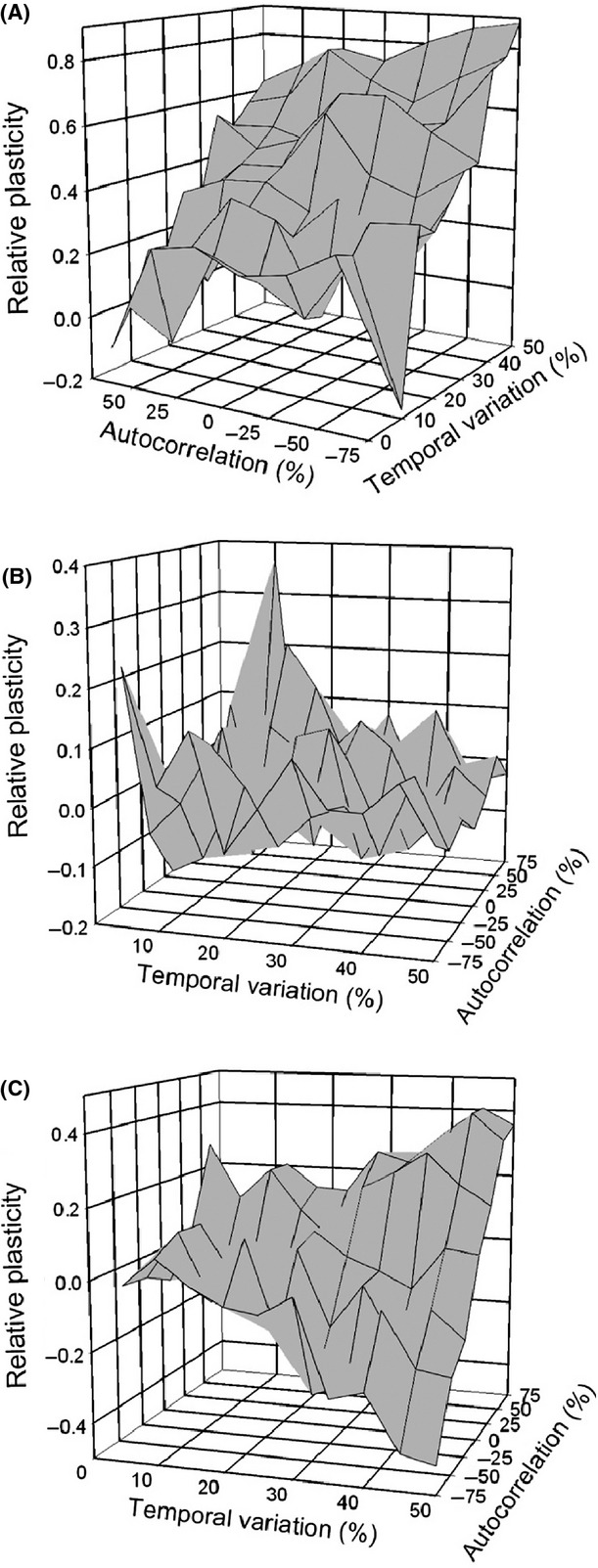
The propensity for plasticity to be favored by temporal variation. A relative plasticity value of 1.0 indicates a pure plasticity outcome. Temporal variation is scaled as a percentage of the standard deviation of that variation. (A) Change once per generation prior to development. (B) Change both before and after development with no correlation for the within-generation change. (C) Change both before and after development with varying magnitudes of positive or negative autocorrelation for the within-generation change. Note the differences in scale of the vertical axes.

When there is a single deme (i.e., no spatial variation), there are two possible patterns of temporal variation: the environment changes once per generation before development occurs, or the environment changes both prior to and after development. In the latter case, the within-generation changes may or may not be correlated. When the environment changed before development only (Fig. 1A), plasticity was favored as the magnitude of among-generation variation increased. When the among-generation correlation was negative, it was more difficult for the population to track the environmental changes through fixed-development genetic changes, and plasticity was more strongly favored.

When the environment changed both before and after development and the within-generation change was uncorrelated (Fig. 1B), plasticity was not favored because the environment of development did not provide a reliable cue for the environment of selection. At most, plasticity was weakly favored when the magnitude of the variation was very low and the correlation among generations was high (back left corner of Fig. 1B). However, the overall selection against plasticity resulted in very noisy values around zero, so any trends in the output should be viewed with skepticism.

When the within-generation change was correlated (Fig. 1C), greater correlations favored plasticity, especially as the magnitude of that variation increased. However, plasticity was not as strongly favored as in the case of among-generation variation only. When the within-generation correlation was positive, selection favored positive reaction norms, and when the correlation was negative, it favored negative reaction norms. The latter has been referred to as countergradient variation when it occurs in a spatial context (Conover and Schultz 1995; Ghalambor et al. 2007), because the reaction norm appears to be counter to an adaptive reaction norm. However, the reaction norm is adaptive because the environment at the time of development is opposite the environment at the time of selection. Such a reaction norm would appear to be maladaptive in a laboratory or greenhouse experiment if attention is not paid to natural patterns of environmental variation.

### Spatial variation and temporal variation before selection (pattern 1)

More complex patterns of adaptation occurred when the environment varied both temporally and spatially. Combining the two sources of heterogeneity allows for two types of temporal variation as experienced by an organism: the organism remains in one place and the environment changes (temporal variation sensu stricto), or the organism moves to a different environment. In these simulations, the effects of spatial variation alone are shown for marginal values of temporal variation of 0.

**Figure 2:**
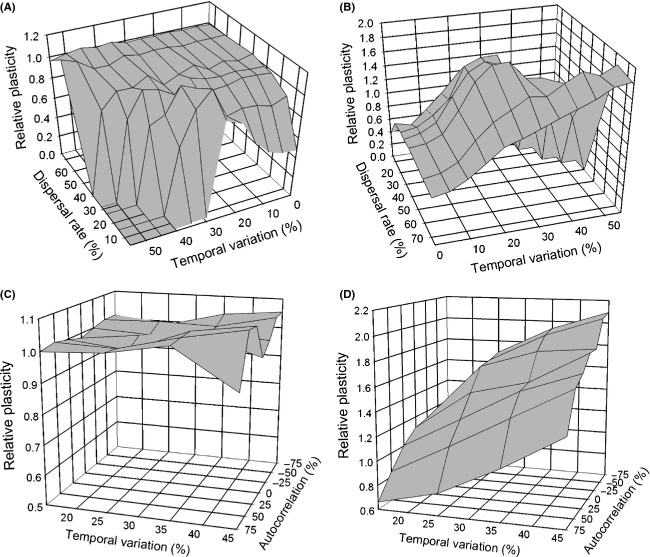
The effect of environmental change after selection only (pattern 1, Table 1) on the evolution of phenotypic plasticity. (A) and (B) The interaction of dispersal rate and the standard deviation of the local phenotypic optima (correlation among generations = 0). (C) and (D) The interaction of temporal variation and among-generation correlation (dispersal rate = 64%). Temporal variation is scaled as a percentage of the length of the environmental gradient. (A) and (C) Selection before dispersal (*select first*); (B) and (D) dispersal before selection (*move first*).

Although verbal arguments sometimes equate these two types of change, the results shown here demonstrate that they are not always equivalent in their propensity to favor plasticity. Consider first the scenario where the environment prior to development is fixed in time but the environment of selection varies among generations (pattern 1, Table 1). [These results appeared previously in Scheiner and Holt (2012) and are replicated here for comparative purposes]. For the *select first* life history pattern, intermediate to high dispersal rates favored plasticity because of the among-generation variation due to dispersal (Fig. 2A). Parameter combinations of low dispersal and high temporal variation led to 100% extinction; for these parameter combinations, relative plasticity is shown as 0. This selection for plasticity was unaffected by correlations in among-generation temporal variation (Fig. 2C).

In contrast, for the *move first* life history pattern, plasticity was favored at intermediate dispersal rates and increasing among-generation temporal variation because the among-generation variation due to dispersal overwhelms the uncertainty caused by within-generation differences in the environment between development and selection (Fig. 2B). Again, parameter combinations of low dispersal and high temporal variation led to 100% extinction. Most notably, very high levels of temporal variation, especially when there was a negative correlation among generations, led to hyperplasticity, a reaction norm slope substantially greater than 1 (Fig. 2D). This hyperplasticity is a form of bet hedging. See Scheiner and Holt (2012) for an explanation of this phenomenon; it will be explored in more detail in a subsequent paper.

In the previous simulations, each deme varied independently in time, while the gradient structure of the environment maintained a spatial correlation. Contrast that scenario with one where the temporal variation is also synchronized in space such that all demes vary in the same direction and to the same magnitude each generation. In this case, even moderate amounts of temporal variation resulted in extinction of the metapopulation for both life history patterns (Fig. S1). In contrast, in the previous simulations, temporal variation was uncorrelated among demes and plasticity buffered against demographic stochasticity (Fig. 2B and D). All of these simulations assume soft selection – each deme returns to its carrying capacity following reproduction if at least one individual survives. The results might differ for hard selection where the per capita reproductive rate is fixed.

### Spatial variation and temporal variation before development (pattern 2)

The temporal variation and dispersal differ in their effects depending on when during the life cycle the temporal variation occurs. For the *select first* life history pattern, in contrast to the previous scenario, when variation occurred before development and the environment at selection was fixed (pattern 2, Table 1), plasticity was disfavored for even low amounts of variation (Fig. S2A). For the *move first* life history pattern, the same result held (Fig. S2B). In neither case did the dispersal effect overcome the fact that the environment at the time of development provided no cue to the environment of selection. For these simulations, a relative plasticity of 0 indicates selection for genetic differentiation, rather than extinction. In contrast, if the temporal variation was synchronized among demes, extinction occurred as described for pattern 1 (results not shown).

### Spatial variation and temporal variation before development carried over to selection (pattern 3, correlation=1)

**Figure 3:**
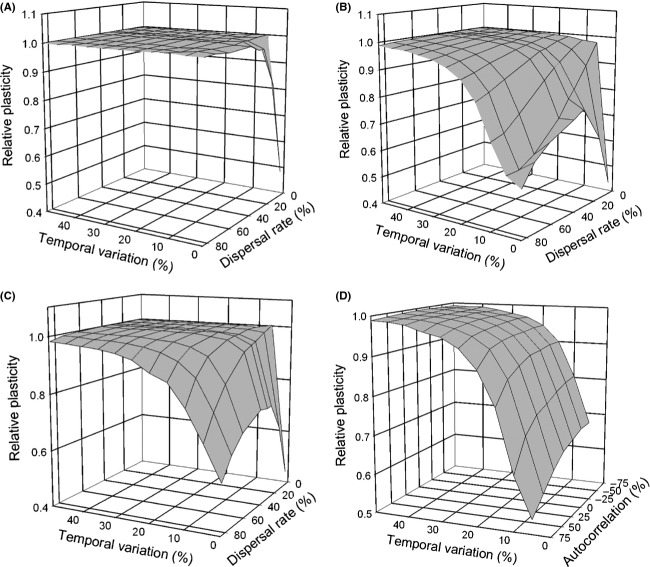
The effect of environmental change before development only that carries over to selection (pattern 3, Table 1, within-generation correlation = 1) on the evolution of phenotypic plasticity. Temporal variation is scaled as a percentage of the length of the environmental gradient. (A) and (B) The interaction of dispersal rate and the standard deviation of the local phenotypic optima (correlation among generations = 0). (A) Selection before dispersal (*select first*); (B) dispersal before selection (*move first*). (C) Change synchronized across the metapopulation (correlation among generations = 0) for dispersal before selection (*move first*). (D) The interaction of temporal variation and among-generation correlation (dispersal rate = 64%) for dispersal before selection (*move first*).

When change before development carries through to selection (pattern 3, correlation=1), the effects of temporal variation and dispersal work synergistically. For the *select first* life history pattern, the optimal reaction norm was favored for even small amounts of temporal variation (Fig. 3A), as is expected because the environment of development provides a strong cue for the environment of selection. This pattern of temporal variation eliminated the extinction effect at high temporal variation and low dispersal rates because individuals that expressed the optimal reaction norm had high fitness under all conditions. This strong favoring of plasticity and lack of extinction were not affected by either spatial synchronization or by among-generation temporal correlations (results not shown).

For the *move first* life history pattern, the uncertainty caused by dispersal between development and selection, reduced selection for plasticity at low to moderate amounts of temporal variation (Fig. 3B). Again, the extinction effect was eliminated. Spatial synchronization of the temporal variation strongly favored plasticity, except at very high dispersal rates (Fig. 3C). Among-generation temporal correlation had a very small effect and only when the magnitude of temporal variation was small (Fig. 3D). Notably, selection for hyperplasticity was eliminated.

### Spatial variation and temporal variation before development and before selection (pattern 3, correlation<1)

**Figure 4:**
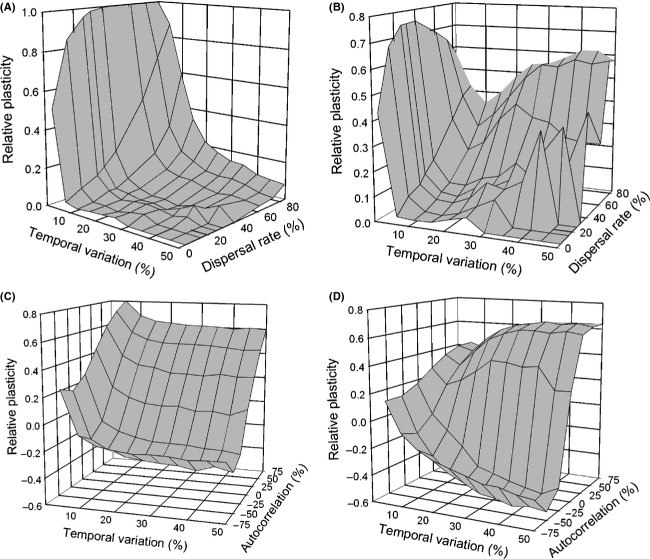
The effect of environmental change both before and after development (pattern 3, Table 1) on the evolution of phenotypic plasticity. (A) and (B) The interaction of dispersal rate and the standard deviation of the local phenotypic optima (correlation within generation = 0). (C) and (D) The interaction of temporal variation and autocorrelated change (dispersal rate = 64%). Temporal variation is scaled as a percentage of the length of the environmental gradient. (A) and (C) Selection before dispersal (*select first*); (B) and (D) dispersal before selection (*move first*).

If environmental change happens both within and between generations (pattern 3, correlation<1), then selection responses are complex and depend on the life history pattern. For the *select first* life history pattern, when those changes were uncorrelated, even moderate amounts of temporal variation selected against plasticity (Fig. 4A). This result is similar to the pattern 2 response (Fig. S2A), except for weakly favoring plasticity at very high dispersal rates. In contrast to pattern 1 where the effect of dispersal overwhelmed the effect of temporal variation, the opposite occurred. When the temporal correlation was not zero, plasticity was favored (Fig. 4C). A positive correlation selected for a positive reaction norm and a negative correlation selected for a negative reaction norm, although the slope of the latter was lower. The magnitude of the temporal variation had little effect on this response.

The most complex pattern is seen for the *move first* life history pattern when the temporal correlation was 0 (Fig. 4B). As with the other life history pattern, increasing temporal variation selected against plasticity. However, when the magnitude of temporal variation was large, high dispersal rates selected for partial plasticity. Under pattern 1, these same conditions selected for hyperplasticity (Fig. 2B). (The very noisy results for high temporal variation and low dispersal rates is due to complete extinction for some parameter combinations.) When the temporal correlation was not zero, the response was similar to the other life history pattern (Fig. 4D), except that the magnitude of the slopes of the negative reaction norms were similar to those of the positive reaction norms. For either life history pattern, the effect of spatial synchronization was the same as pattern 1 (Fig. S2), extinction at even small magnitudes of temporal variation (results not shown).

### Island versus stepping-stone migration pattern

**Figure 5:**
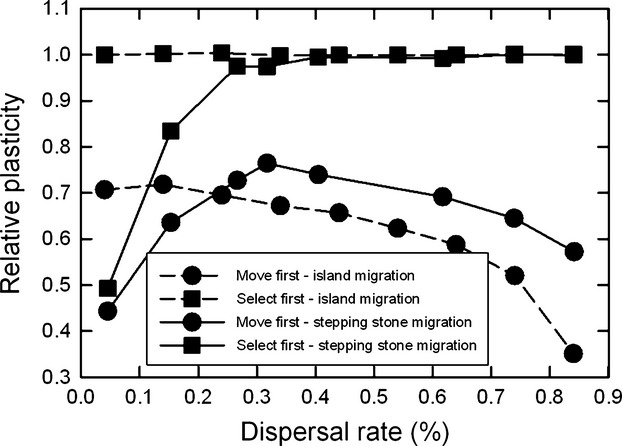
The effect of pattern of dispersal pattern (island migration vs. stepping-stone migration) on the evolution of phenotypic plasticity. There is no temporal environmental variation.

For spatial variation only and the *select first* life history pattern, island migration increased selection for plasticity at low dispersal rates (Fig. 5) because it enhanced the among-generation variation created by dispersal. For the *move first* life history pattern, island migration similarly increased selection for plasticity at low dispersal rates, but selected for lower amounts of plasticity at high dispersal rates. This decrease occurred because island dispersal enhanced the uncertainty due to the potential for more distant dispersal after development but before selection.

These effects also held when there was among-generation variation prior to selection (pattern 1). There was still selection for hyperplasticity, but the magnitude of the effect was smaller (compare Figs 2B and D, S3A,B). For pattern 1, island migration diminished the extinction region for both life history patterns. When the variation was spatially synchronized, for the *move first* life history pattern, extinction occurred at about a 30% magnitude of temporal variation (results not shown), as opposed to about 15% for stepping-stone migration (Fig. S1B). There was no difference in the extinction pattern for the *select first* life history pattern.

For pattern 3, when the correlation was less than 1, island migration strongly favored plasticity (Fig. S4) in contrast to stepping-stone migration (Fig. 4). For the *select first* life history pattern, the island migration selection response was similar to the response for stepping-stone migration when the within-generation temporal correlation was 1 (Fig. 3A). For the *move first* life history pattern, the island migration selection response was similar to the stepping-stone migration response when there was spatial synchronization (Fig. 3C). Because island migration allows individuals to move to any deme with equal probability, from the perspective of an evolutionary lineage, temporal and spatial variation become more synchronized or correlated.

## Discussion

The fundamental question that we are presented by the theory of the evolution of phenotypic plasticity (Appendix) is why adaptive plasticity is not more common. In this paper, I address one key constraint on that evolution, the pattern of environmental heterogeneity. Environmental heterogeneity is a necessary component for the evolution of phenotypic plasticity (theory proposition 1). However, because the pattern of that variability in space and time determines cue reliability, the pattern is critical for determining when plasticity will be favored by natural selection (theory proposition 4).

### Cue reliability – external constraints

Cue reliability lies at the intersection of internal and external constraints. Reliability has two components. The first component is environmental: the correlation between the environment at the time that the phenotype is determined and the time that selection occurs. That correlation can be complex and need not be the same environmental cause at each moment. For example, cool temperatures at one time of year may signal fewer herbivores later in time. The causal chain between temperature and herbivore abundance may be complex, but that complexity gets collapsed into the correlation, that is, cue reliability.

In my model, external constraints on cue reliability are manifested in several ways. Consider the case of a single deme with temporal variation (Fig. 1). When the environment varies only among generations, the cue is highly reliable and selection for plasticity is determined by the magnitude and pattern of environmental heterogeneity. When the environment also varies between development and selection, then the reliability of the cue depends on the environmental correlation.

Spatial variation adds complexity. Now cue reliability is a function of both the pattern of temporal variation and the pattern of dispersal. Consider the case of the *move first* life history pattern with no temporal variation and a stepping-stone migration pattern. Movement along a gradient inherently includes a component of spatial autocorrelation in the environment. Thus, increasing dispersal rates from low to intermediate increases selection for plasticity (Fig. 5) because environmental variability is increasing in a reliable fashion. As dispersal rates (and thus distances) continue to increase, however, plasticity is less favored because cue reliability decreases. Plasticity is more strongly favored at low dispersal rates by the island migration pattern because the increase in environmental heterogeneity experienced among generations offsets the decrease in cue reliability. For the *select first* life history pattern, the cue is always reliable, thus the strong evolutionary response by phenotypic plasticity.

### Cue reliability – internal constraints

Developmental constraints are the second component of cue reliability. The time that it takes for the phenotype to respond to the cue sets the bounds on the temporal correlation of the environment. If the phenotype can respond very quickly (e.g., color change by a chameleon), then cue reliability will always be very high. In contrast, slow responses (e.g., changes in leaf toughness in response to herbivory that occurs by the production of a new set of leaves) create the potential for a poor correlation and low cue reliability.

In my model, developmental delays are represented by the intragenerational environmental correlation, the change in the environment between the time that the phenotype is set and when selection happens (pattern 3, Table 1; Fig. 4). For the *select first* life history pattern, very little variation is necessary to disfavor plasticity when the correlation is zero and the cue is unreliable, but that effect is offset by an increase in the environmental correlation and cue reliability. For the *move first* life history pattern, cue unreliability is offset by high dispersal rates. Just as with pattern 1 favoring hyperplasticity with the *move first* life history pattern (Fig. 2B), the same effect is happening here. The high dispersal rate combined with the large temporal variation means that a given lineage is likely to experience a wide range of environmental heterogeneity. Plasticity is then favored because it increases the phenotypic range of that lineage as a form of bet hedging. Thus, developmental constraints have to be considered within the overall life history pattern of the organism.

### The ecology of the organism

Many studies of organismal plasticity fail to consider the ecology of the organism. By this, I mean that data interpretation is made in the context of neither short-term nor long-term patterns of environmental heterogeneity of the source population. Laboratory or greenhouse experiments often involve organisms from one or more populations or species that are raised in temporally fixed environments. The first error is not considering whether that environmental factor is fixed in nature, or whether it varies daily, weekly, seasonally, and so forth. Even when our interest is in nonplastic traits, we know that ignoring such variation can lead to incorrect conclusions (e.g., Paaijmans et al. 2010). This problem is likely to be magnified for plastic traits, although it has been less explored (DeWitt and Scheiner 2004).

The second error is making assumptions about whether plasticity will be favored in one or another population or species by just considering one aspect of environmental heterogeneity. For example, two populations may differ in the magnitude of temporal variation, while having similar magnitudes of spatial heterogeneity. Or the researcher may consider only among-year variation while ignoring short-term variation within the context of developmental timing and cue reliability.

Most published papers on plasticity fail to provide any information about the temporal and spatial pattern and magnitude of environmental heterogeneity of the source location(s). Because most such studies are examining the outcome of selection by measuring plasticity under nonfield conditions, interpreting the results is impossible without such a context.

The ecology of the organism also must be considered within the context of the biology of the organism. A failure to consider the timing of developmental cues and the length of developmental delays may lead to conclusions of maladaptive plasticity. For example, increasing root mass relative to shoot mass is generally adaptive under conditions of low water. Consider a situation where rainfall in one month is negatively correlated with rainfall in the next month and it takes 3 weeks to grow new roots. In that situation, wet conditions might provide a cue for increasing root growth. A naïve investigator who simply observed that wetter conditions in the greenhouse led to greater relative root mass would conclude that such plasticity was maladaptive, when consideration of the ecological context would conclude that it was adaptive.

### Space versus time

Many discussions of environmental heterogeneity assume that variation in space is substitutable for variation in time. As shown here, that is not true. Temporal variation alone selects for lower amounts of plasticity than spatial variation alone (compare Figs 1A, 5). Temporal and spatial variation do not combine additively but depend on the life history pattern (compare Figs 2A and B, 4A and B). The pattern of evolutionary response can be quite complex when individuals move in space after development but before selection. In contrast, when the environment changes in time after development, plasticity is simply disfavored. Variation in time and variation in space have different effects on environmental variation and cue reliability. All types of variation are not alike.

### Robustness of conclusions

The results presented here provide predictions about equilibrium outcomes. Transient effects can be quite different and those transients can last for hundreds of generations (Scheiner and Holt 2012; Scheiner et al. 2012). Thus, these results should be interpreted as general information about what conditions favor plasticity over genetic differentiation, and not necessarily be used to make precise predictions about particular situations. To make specific predictions, these models should be used as guidelines for building more specific models. Most important, researchers need to be cognizant of not just current conditions, but also the long-term history of any population or species. The results of these models might provide predictions for large-scale patterns, such as a comparison of species across an entire clade (e.g., Hammond et al. 2012) or within a species across an entire continent (e.g., Samis et al. 2008) because such comparisons subsume much of the idiosyncratic variation among populations or species.

The dispersal patterns examined here – the stepping-stone and island migration patterns – represent the ends of a continuum from high to low spatial autocorrelation, and both patterns converge on panmixia at very high dispersal rates. Thus, the results shown in this paper likely bracket the outcomes found in nature.

The results in this paper provide a context for evaluating other models that consider more limited conditions such as assuming just temporal or spatial variation. Nearly all other models with spatial variation assume a propagule pool or an island migration pattern. As island migration more strongly favors phenotypic plasticity (Figs 4, S4), those models were likely to find that plasticity was favored over a broad parameter space.

## Conclusion

All models trade off generality, precision, and realism (Levins 1966). The models in this paper are meant to be general, but are also an attempt to increase the realism of plasticity models by considering a wider range of types and combinations of environmental heterogeneity. As such they provide guidance to future modeling efforts by indicating which factors, alone or in combination, may be fruitful for future explorations.

My results provide a partial resolution to the question of why adaptive plasticity is less common than we would expect by logical considerations alone. These models show that cue reliability can be an important constraint on selection for plasticity (theory proposition 4). More information on actual cue reliability and the ecological and developmental context of trait plasticity is needed before we can answer this question. We know that selection for plasticity is constrained. What we do not yet know is the relative importance of external and internal constraints.

## Appendix

Although many papers have been written about the evolution of phenotypic plasticity and its theoretical underpinnings (Berrigan and Scheiner 2004), none of them have made explicit the propositions of that theory. As part of my ongoing efforts to strengthen the use of theory in ecology and evolution, I list those propositions. The theory of plasticity evolution as here articulated is a constitutive theory within the theory of evolution, and this paper delineates a set of models within that theory. For an explanation of this theory hierarchy, see Scheiner (2010) and Scheiner and Willig (2011).

A constitutive theory is encapsulated by its domain and propositions. The domain of this theory is evolutionary change in trait plasticity, that is, the evolutionary response of trait plasticity to natural selection. The domain does not include the effect of phenotypic plasticity on other evolutionary processes or outcomes (e.g., speciation, Schlichting 2004). The six propositions of the theory of the evolution of phenotypic plasticity are:

1. Environmental heterogeneity exists that affects the phenotypic expression of traits.
2. The optimal phenotypic value of these plastic traits varies in space and/or time.
3. These plastic traits meet the conditions required for evolution by natural selection.
4. Nonoptimal plasticity may result because the environment at the time that the phenotype is determined does not provide a reliable cue about the environment at the time of selection.
5. Nonoptimal plasticity may result from maintenance, production or information-acquisition costs of plasticity.
6. Nonoptimal plasticity may result from developmental limitations on plasticity.

The first three propositions define the minimal conditions for natural selection of plasticity. Those conditions are the same as for natural selection of any trait plus the requirement for environmental heterogeneity. The other propositions are the conditions that subsequently cause restrictions on the evolution of optimal plasticity by imposing various costs and limitations. Proposition 4 addresses external factors; propositions 5 and 6 address internal factors. More information about costs and limitations of plasticity can be found in DeWitt et al. (1998).

## Supporting Information

**Figure S1.** The interaction of dispersal rate and the standard deviation of the local phenotypic optima on the evolution of phenotypic plasticity when the environmental change occurs after selection only (pattern 1, Table 1) and when that change is synchronized across the metapopulation (correlation among generations = 0). Values of relative plasticity of 0 indicate that the metapopulation went extinct in all 60 replicates. Temporal variation is scaled as a percentage of the length of the environmental gradient. (A) Selection before dispersal (*select first*); (B) dispersal before selection (*move first*).

**Figure S2.** The interaction of dispersal rate and the standard deviation of the local phenotypic optima on the evolution of phenotypic plasticity when the environmental change occurs before development only (pattern 2, Table 1) (correlation among generations = 0). Temporal variation is scaled as a percentage of the length of the environmental gradient. (A) Selection before dispersal (*select first*); (B) dispersal before selection (*move first*).

**Figure S3.** The effect of the island migration pattern on the evolution of phenotypic plasticity when the environmental changes after development only (pattern 1, Table 1) and dispersal occurs before selection (*move first*). (A) The interaction of dispersal rate and the standard deviation of the local phenotypic optima (correlation among generations = 0). (B) The interaction of temporal variation and among-generation correlation (dispersal rate = 64%). Temporal variation is scaled as a percentage of the length of the environmental gradient.

**Figure S4.** The effect of the island migration pattern on the evolution of phenotypic plasticity when the environmental changes both before and after development (pattern 3, Table 1). The interaction of dispersal rate and the standard deviation of the local phenotypic optima (correlation within generation = 0). (A) Selection before dispersal (*select first*); (B) dispersal before selection (*move first*).
