# Supplementary material for: The genetics of phenotypic plasticity. XII. Temporal and spatial heterogeneity
Source: Ecol Evol. 2013 Oct 22;3(13):4596–609. doi: 10.1002/ece3.792 (PMC3856757; doi:10.1002/ece3.792)
Supplement: Supplementary file 1 [file ece30003-4596-SD1.docx]

Figure S1. The interaction of dispersal rate and the standard deviation of the local phenotypic optima on the evolution of phenotypic plasticity when the environmental change occurs after selection only (pattern 1, Table 1) and when that change is synchronized across the metapopulation (correlation among generations = 0). Values of relative plasticity of 0 indicate that the metapopulation went extinct in all 60 replicates. Temporal variation is scaled as a percentage of the length of the environmental gradient. (A) Selection before dispersal (*select first*); (B) dispersal before selection (*move first*).

Figure S2. The interaction of dispersal rate and the standard deviation of the local phenotypic optima on the evolution of phenotypic plasticity when the environmental change occurs before development only (pattern 2, Table 1) (correlation among generations = 0). Temporal variation is scaled as a percentage of the length of the environmental gradient. (A) Selection before dispersal (*select first*); (B) dispersal before selection (*move first*).

Figure S3. The effect of the island migration pattern on the evolution of phenotypic plasticity when the environmental changes after development only (pattern 1, Table 1) and dispersal occurs before selection (*move first*). (A) The interaction of dispersal rate and the standard deviation of the local phenotypic optima (correlation among generations = 0). (B) The interaction of temporal variation and among-generation correlation (dispersal rate = 64%). Temporal variation is scaled as a percentage of the length of the environmental gradient.

Figure S4. The effect of the island migration pattern on the evolution of phenotypic plasticity when the environmental changes both before and after development (pattern 3, Table 1). The interaction of dispersal rate and the standard deviation of the local phenotypic optima (correlation within generation = 0). (A) Selection before dispersal (*select first*); (B) dispersal before selection (*move first*).
